# AAV1.NT‐3 gene therapy in the SOD1KO mouse model of accelerated sarcopenia

**DOI:** 10.1002/jcsm.13303

**Published:** 2023-08-08

**Authors:** Lingying Tong, Burcak Ozes, Kyle Moss, Morgan Myers, Alicia Ridgley, Zarife Sahenk

**Affiliations:** ^1^ Center for Gene Therapy The Abigail Wexner Research Institute, Nationwide Children's Hospital Columbus OH USA; ^2^ Department of Pediatrics and Neurology Nationwide Children's Hospital and The Ohio State University Columbus OH USA; ^3^ Department of Pathology and Laboratory Medicine Nationwide Children's Hospital Columbus OH USA

**Keywords:** Aging, Gene therapy, mTORC, NT‐3, Sarcopenia, SOD1KO

## Abstract

**Background:**

Sarcopenia, an age‐related loss of muscle mass, is a critical factor that affects the health of the older adults. The SOD1KO mouse is deficient of Cu/Zn superoxide dismutase, used as an accelerated aging model. We previously showed that NT‐3 improves muscle fibre size by activating the mTOR pathway, suggesting a potential for attenuating age‐related muscle loss. This study assessed the therapeutic efficacy of AAV1.NT‐3 in this accelerated aging model.

**Methods:**

Twelve 6 months old SOD1KO mice were injected intramuscularly with a 1 × 10^11^ vg dose of AAV1.tMCK.NT‐3, and 13 age‐matched SOD1KO mice were used as controls. The treatment effect was evaluated using treadmill, rotarod and gait analyses as well as histological studies assessing changes in muscle fibre, and fibre type switch, in tibialis anterior, gastrocnemius, and triceps muscles, and myelin thickness by calculating G ratio in sciatic and tibial nerves. Molecular studies involved qPCR experiments to analyse the expression levels of mitochondrial and glycolysis markers and western blot experiments to assess the activity of mTORC1 pathway.

**Results:**

Treatment resulted in a 36% (154.9 vs. 114.1; *P* < 0.0001) and 76% increase (154.3 vs. 87.6; *P* < 0.0001) in meters ran, with treadmill test at 3 and 6 months post gene delivery. In addition, the treated cohort stayed on rotarod 30% (52.7 s vs. 40.4 s; *P* = 0.0095) and 54% (50.4 s vs. 32.7 s; *P* = 0.0007) longer, compared with untreated counterparts at 3 and 6 months post injection. Gait analysis, performed at endpoint, showed that stride width was normalized to wild type levels (29.3 mm) by an 11% decrease, compared with untreated cohort (28.6 mm vs. 32.1 mm; *P* = 0.0014). Compared with wild‐type, SOD1KO mice showed 9.4% and 11.4% fibre size decrease in tibialis anterior and gastrocnemius muscles, respectively, which were normalized to wild type levels with treatment. Fibre diameter increase was observed prominently in FTG fibre type. G ratio analysis revealed hypomyelination in the tibial (0.721) and sciatic (0.676) nerves of SOD1KO model, which was reversed in the NT‐3 cohort (0.646 and 0.634, respectively). Fibre size increase correlated with the increase in the p‐S6 and p‐4E‐BP1 levels, and in the glycolysis markers in tibialis anterior. Alterations observed in the mitochondrial markers were not rescued with treatment. Overall, response to NT‐3 was subdued in gastrocnemius muscle.

**Conclusions:**

This study shows that AAV1.NT‐3 gene therapy protected SOD1KO mouse from accelerated aging effects functionally and histologically. We further confirmed that NT‐3 has potential to activate the mTOR and glycolytic pathways in muscle.

## Introduction

Aging in humans is accompanied by a significant reduction in muscle mass/function as the primary cause of sarcopenia and loss of independence in the elderly.^1^ While the aetiology of sarcopenia is not fully deciphered, among the several contributing factors, oxidative stress with mitochondrial dysfunction has been implicated as a major underlying cause of age‐related muscle loss.^2^, ^3^, ^4^, ^5^ The mouse model that lacks the antioxidant enzyme Cu/Zn superoxide dismutase1 (SOD1KO) is characterized by high levels of oxidative damage in muscle, with an acceleration of sarcopenia.^6^, ^7^, ^8^, ^9^ Studies have demonstrated that SOD1KO mice have a significantly lower muscle mass than wild type (WT) mice starting as early as 3 to 4 months of age, which shows a dramatic acceleration after 6 months; by 20 months, the hindlimb muscle mass is nearly 50% lower than in the age‐matched WT mice.^6^, ^10^ In addition, with increasing age, a progressive motor axonopathy, primarily affecting axon terminals at the neuromuscular junction (NMJ), has been described.^11^ Preferential denervation of fast twitch muscles, beginning between 1 and 4 months of age with relative sparing of slow twitch muscle, has also been reported.^12^ Our previous in vivo and in vitro studies have shown that neurotrophin 3 (NT‐3) increases muscle fibre diameter through direct activation of the mammalian target of rapamycin, mTORC1 pathway.^13^ In previous studies, using NT‐3 gene therapy approach, we illustrated improvements in muscle fibre size in neurogenic, as well as myopathic muscles.^14^, ^15^, ^16^ This approach ensures systemic NT‐3 effect through secretion from muscle into circulation. In addition, NT‐3 was shown to improve hypomyelination state and NMJ connectivity.^14^, ^15^, ^16^ Moreover, we recently reported that NT‐3 gene therapy attenuated age‐related musculoskeletal changes and improved function in a naturally occurring sarcopenia model, in aged WT C57BL/6J mice^17^. In this study, we explored potential application of NT‐3 gene therapy for muscle wasting in the SOD1KO mice, an accelerated model for sarcopenia characterized by high levels of oxidative damage.

As done previously, we used self‐complimentary adenoassociated virus serotype 1 (scAAV1) vector, and triple tandem repeat muscle‐specific creatine kinase (tMCK) promoter to assess the therapeutic efficacy of NT‐3 in SOD1KO mice via intramuscular (IM) delivery of scAAV1.tMCK.NT‐3 at 6 months of age; a time where sarcopenic changes are already occurring.^6^, ^18^, ^19^ Quantitative histopathology in muscle and peripheral nerves and functional studies assessing motor strength were used as outcome measures for evaluation of the efficacy of NT‐3 gene therapy in this model. In addition, to further our understanding of the NT‐3 effect on oxidative stress induced‐sarcopenia, we assessed the mitochondria biogenesis, oxidative phosphorylation, mTORC1 activation and glycolysis biomarkers in muscle. The results show that AAV1.NT‐3 gene therapy in SOD1KO mice unequivocally improved the function of sarcopenic muscle and muscle fibre size, which was most prominent for fast twitch glycolytic (FTG) fibres, along with increased expression of glycolytic pathway markers.

## Methods

### Animals and treatment groups

The SOD1KO mouse used in the study (JAX stock #002972) was previously defined as a model for accelerated aging and frailty.^19^ Genotypes of SOD1KO mice obtained from breeding pairs were verified by PCR analysis of genomic DNA isolated from tail clips. All animal experiments were performed according to the guidelines approved by The Research Institute at Nationwide Children's Hospital Animal Care and Use Committee that operates full accordance with the Animal Welfare Act and the Health Research Extension Act (IACUC approval number: AR18‐00076).

Six‐month‐old SOD1KO (6 males and 6 females, *n* = 12) mice received 1 × 10^11^ vg dose of AAV1.tMCK.NT‐3 via IM injection into the gastrocnemius muscle. Age‐ and sex‐matched SOD1KO (7 males and 6 females, *n* = 13) mice were injected with Ringer's lactate as controls. The mice were sacrificed 6 months post‐injection. Functional status of mice was monitored using treadmill, rotarod and gait analysis, and mice were euthanized with a xylazine/ketamine cocktail, and blood, sciatic nerves and selected muscles from front and hind limbs were collected at 6 months post gene injection.

### Recombinant AAV.NT‐3 vector production and potency

Design of scAAV vector with serotype 1 containing NT‐3 with the muscle specific tMCK promoter was described previously and produced in our Viral Vector Core at Nationwide Children's Hospital, Columbus.^14^ Aliquots of virus were kept at −80°C until used. Terminal blood samples from anaesthetized treated and untreated cohorts were collected by cardiac puncture, and serum was assayed for NT‐3 levels using a capture ELISA as previously reported.^14^

### Run to exhaustion test

Mice were exercised to exhaustion via treadmill (Columbus Instruments, Exer‐6 M Treadmill), according to a previously described protocol.^20^ The treadmill was operated at a 4–5° decline. Mice were acclimated to the treadmill for 3 days before data collection, with 10‐min‐long 7–10 m/min runs each day. Mice were run to exhaustion with increasing treadmill speed by 1 m/min each minute, starting at an initial 7 m/min velocity in lanes with a shock plate that pulses at a frequency of ~3 Hz. Mice were considered ‘exhausted’ when they were unable to re‐engage the treadmill for 3 s after resting on the shock‐plate. Run duration was recorded and used to calculate the distance ran.

### Rotarod

Mouse motor function and balance was tested at baseline and endpoint by using the accelerating rotarod (Columbus Instruments, Ohio, USA).^14^ Mice were trained on the rotarod apparatus for 2 weeks to acclimate to testing protocol prior to data collection. The protocol was run at 5 rpm with a constant acceleration of 0.5 rpm/s, and the average of the best two‐out‐of‐three trials was included.

### Gait analysis

Stride width of the SOD1KO mice were tested at 6 months post‐injection. A 3‐cm‐wide by 28‐cm‐long white paper was placed at the bottom of a long narrow corridor. Mice walked along the corridor after their hind feet were coated with animal safe ink (ReignDrop Washable Ink). The centres of the footprints were marked on the paper. Two lines, one connecting the right footsteps and the other one connecting the left footsteps were drawn. To get the best representation of the data, four to six random locations were chosen for each mouse and the distance between the left and right footsteps were measured at these locations. The average of the distances was calculated as stride width of the relevant mouse.

### Histological analysis

Tibialis anterior, gastrocnemius, and triceps muscles from treated and untreated mice were collected and 12 μm thick cross cryostat‐sections were cut. Succinic dehydrogenase (SDH) enzyme histochemistry was performed as previously described^20^ to assess metabolic fibre type distribution and myofibre size changes in the aging muscle. One representative area from deep, intermediate, and superficial zones of the muscles were photographed at 20× magnification using an Olympus BX41 microscope and SPOT Insight 12Mp sCMOS camera. Shortest distance across the muscle fibre was measured as fibre diameter (Zeiss Axiovision LE4 software V4.9.1.0) and mean fibre diameter (mean ± SEM) was calculated for each fibre type (STO, FTO and FTG) as well as for combination of all fibre types. Fibre type percent distribution of total fibres was determined for each mouse from each treatment group. Data was obtained from a total of 1761 (*n* = 6), 1852 (*n* = 6) and 3254 (*n* = 11) fibres of the treated cohort, and 2059 (*n* = 6), 1722 (*n* = 6) and 2460 (*n* = 7) fibres of the untreated cohort for tibialis anterior, triceps and gastrocnemius muscles, respectively.

G ratio was calculated to assess the myelin thickness of fibres in sciatic and tibialis nerves, as described previously.^15^ Semithin, toluidine blue‐stained cross sections were prepared from sciatic (*n* = 6) and tibial (*n* = 4) nerves of both treated and untreated SOD1KO mice, and age matched WT control mice, with even sex distribution. Three randomly selected nonoverlapping areas were photographed at 100× magnification using an Olympus BX41 microscope and SPOT Insight 12Mp sCMOS camera. Myelin interior and exteriors were outlined in Axiovision (AxioVs40x64 V 4.9.1.0) to determine the area, which was used to calculate diameters to determine g ratio. A total of 2834 (sciatic) and 1839 (tibial) fibres for treated, 3377 (sciatic) and 1982 (tibial) for untreated and 1658 (tibial) for 1‐year‐old control mice were measured to generate scattergrams and the percent g ratio distribution histograms. Slopes of treated versus untreated and 1‐year‐old versus 2‐year‐old mice were compared using GraphPad Prism (9.0.0).

### Protein extraction and western blot analysis

Twenty micrometre thick sections from frozen tibialis anterior and gastrocnemius muscle blocks (20 section per block, *n* = 3 per group) were put into 2 mL centrifuge tubes and homogenized in homogenization buffer [125 mM Tris–HCL pH 6.8, 4% SDS, 4 M Urea solution with 1× Halt protease inhibitor (ThermoFisher) and 1× phosphatase inhibitor (Sigma)] using a disposable pestle. The lysate was then incubated on a rotary spin cycle at 4°C for 2 h, followed by centrifugation at 10 000 *g* for 10 min at 4°C. The supernatant was then transferred to a new tube. Protein samples were run in Novex 10 to 20% Tricine mini protein gel (ThermoFisher) and transferred to PDVF membranes (GE Healthcare). Membranes were blocked for 1 h at room temperature with 5% milk in TBS‐T (TBS buffer with 0.05% Tween‐20). Primary antibodies were then added to membranes in TBS‐T buffer overnight at 4°C. TBS‐T was used to wash the membranes for 5 min, three times, and then membranes were incubated with secondary antibodies in 5% milk in TBS‐T for 1 h, then washed with TBS‐T 3 additional times and TBS 2 times, with 5 min each wash. ECL WesternSure premium chemiluminescent substrate (LI‐COR) was used for band detection followed by exposure using Chemidoc Imaging system (Bio‐rad) and band intensities were quantified using ImageJ (NIH). Primary antibodies: anti‐phospho S6 protein Ser235/236 (#4858), anti‐phospho 4EBP1 thr37/46 (#2855) were from Cell Signaling Technology and Anti‐actin antibody (sc‐47778) was from Santa Cruz; secondary antibody: HRP‐linked anti‐rabbit/mouse IgG (#7074/7076) was purchased from Cell Signaling Technology.

### RNA isolation and mRNA quantification

Total RNA was extracted from frozen muscle blocks (20 μm thick sections, 20 section per block) using Mini RNeasy Plus Universal Kit (Qiagen). cDNAs were synthesized using ProtoScript II First Strand cDNA Synthesis Kit (BioLabs). Primer sets (synthesized by IDT) for *Pgc‐1α*, *Cox1*, *Cox3* and *Atp5d* were obtained from previous publications^21^, ^22^, ^23^ and new primers were designed for *Pfkm* (*F‐GAAGATACCAACTCGGACCAC*, *R‐*ATGACCCATGAAGAGCATCA); *Hk‐1* genes (*F‐CGGAATGGGGAGCCTTTGG*, *R‐GCCTTCCTTATCCGTTTCAATGG*); LDHA (F‐CCCTTGAGTTTGTCTTCCATGA, R‐CTCCCCAGAACAAGATTACAGT); MLXipl (F‐TTGTTCAGCCGGATCTTGTC, R‐CACCTCTTCGAGTGCTTGAG); GLUT4 (F‐GTAACTTCATTGTCGGCATGG, R‐AGCTGAGATCTGGTCAAACG). All qPCR was performed using PowerUp SYBR Green Master Mix (ThermoFisher) according to the manufacturer's instructions. qPCR assays were performed using QuantStudio 6 Flex (Applied Biosystems). Expression data were normalized to mouse *Gapdh* mRNA level and data were analysed by ∆∆Ct method. *Gapdh* mRNA levels between groups were comparable.

### Statistics

GraphPad Prism 9.0.0 software was used for all statistical analyses. Depending on the data, unpaired *t*‐test, one‐way ANOVA with Tukey's multiple comparison test, two‐way ANOVA with Sidak's, Tukey's, or Bonferroni's multiple comparison test, or linear regression analysis were performed. The tests that meet the best assumptions of the data were chosen and the name of the statistical analysis performed was given in the figure legends. Significance level was set at *P* < 0.05.

## Results

### Recombinant AAV.NT‐3 vector production and potency

scAAV1.tMCK.NT‐3 design (*Figure* S1A) and production followed previously described methods at Nationwide Children's Hospital, Columbus.^14^ scAAV1.tMCK.NT‐3, at 1 × 10^11^ vg dose was delivered to the gastrocnemius muscle of SOD1KO mice at 6 months of age; blood samples from terminally‐anaesthetized treated and untreated mice were collected by cardiac puncture at 6 months post gene injection and serum was assayed for NT‐3 levels using a capture ELISA as previously reported.^14^ These serum levels (*Figure* S1B) were correlating with functional and histologic outcome measures as described.

### Neurotrophin‐3 gene therapy improves function in accelerated aging model SOD1KO mouse

**Figure 1:**
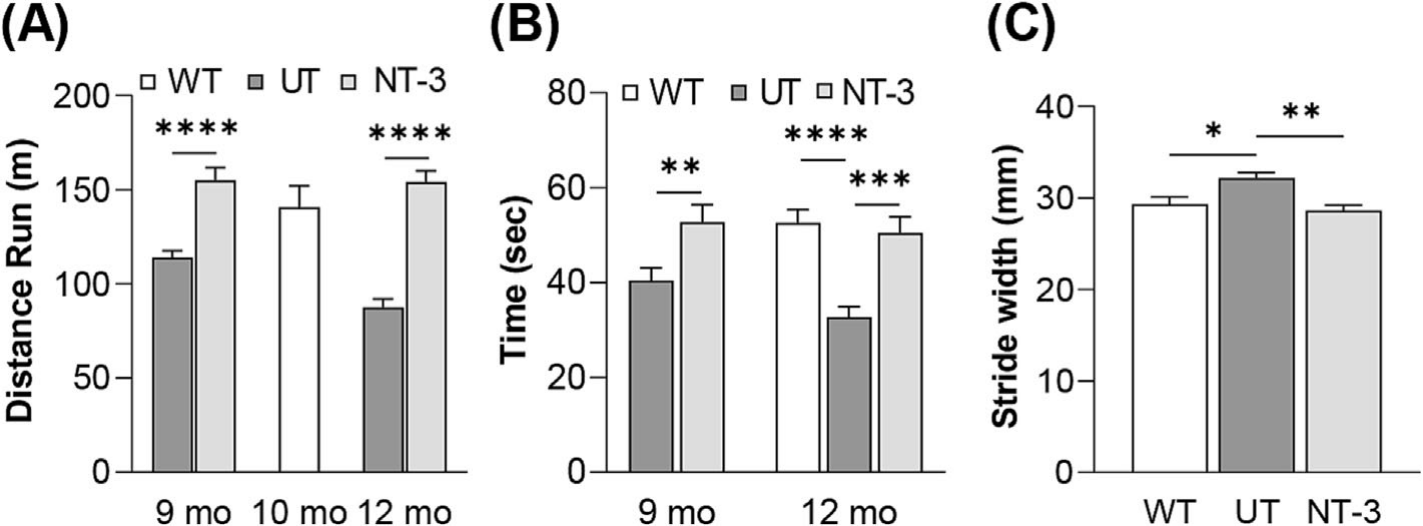
NT‐3 treatments protects function in SOD1KO mice. (A) Treadmill test showed significant improvement at 3 and 6 months post‐injection when mice are 9 months and 12 months old (mo), respectively (9 mo, NT‐3: 154.9 m, *n* = 12 vs. UT: 114.1 m, *n* = 13, ****P < 0.0001; 12 mo, NT‐3: 154.3 m, *n* = 11 vs. UT: 87.6 m, *n* = 13, *P* < 0.0001, two‐way ANOVA, Sidak's multiple comparisons test; 10 months old WT mice: 104.8 m). (B) Rotarod analysis revealed treated mice performed significantly better than the untreated mice at 9 months and 12 months old (9 mo, NT‐3: 52.71 s, *n* = 12 vs. UT: 40.42 s, *n* = 12, **P = 0.0095; 12 mo, NT‐3: 50.44 s, *n* = 9 vs. UT: 32.73 s, *n* = 11, ***P = 0.0007, two‐way ANOVA, Sidak's multiple comparisons test; 12 months old WT mice: 52.61 s). (C) Untreated mice had significantly wider strides compared with age‐matched wild type (WT) mice, which stayed in the normal range in the treated cohort (NT‐3: 28.60 mm, *n* = 10; UT: 32.13 mm, *n* = 13; WT: 29.33 mm, *n* = 6; *P = 0.0321, **P = 0.0014, one‐way ANOVA, Tukey's multiple comparisons test).

Run to exhaustion treadmill test was used to assess the functional efficacy of NT‐3 gene therapy. Gene therapy improved treadmill performance at 3 and 6 months post gene delivery, leading to a 36% increase (NT‐3, 154.9 ± 6.7, *n* = 13 vs. UT, 114.1 ± 3.5, *n* = 12; *P* < 0.0001) and 76% increase in running distances until exhaustion (NT‐3, 154.3 ± 5.7, *n* = 13 vs. UT, 87.6 ± 4.5, *n* = 11; *P* < 0.0001) compared with the untreated cohort at 3 and 6 months post injection without significant difference in sexes (*Figure* 1A).

Another functional outcome, rotarod test was carried out at a midpoint (3 months) and endpoint, 6 months post AAV1.NT‐3 gene delivery in SOD1KO mice. Analysis at both times points revealed that NT‐3 gene therapy significantly improved motor coordination in SOD1KO mice (midpoint: NT‐3, 52.7 ± 3.7, *n* = 12 vs. UT, 40.4 ± 2.7, *n* = 12; *P* = 0.0095; endpoint: NT‐3, 50.4 ± 3.4, *n* = 9 vs. UT, 32.7 ± 2.2, *n* = 11; *P* = 0.0007), corresponding to a 30% and 54% increase, respectively (*Figure* 1B). In addition, we assessed if accelerated aging had an effect on the gait pattern of the SOD1KO mice. Gait analysis was conducted on the untreated/Ringer's lactate and AAV1.NT‐3 injected cohorts at endpoint, 6 months post‐gene injection. The stride width of age‐matched wild type mice was 29.3 ± 0.8 mm which was increased to 32.1 ± 0.6 mm in SOD1KO mice (WT, *n* = 6 vs. UT, *n* = 13; *P* = 0.032). NT‐3 gene therapy decreased the stride width by 11%, normalizing the gait of the SOD1KO mice (NT‐3, 28.6 ± 0.6, *n* = 13 vs. UT, 32.1 ± 0.6, *n* = 13; *P* = 0.0014) (*Figure* 1C). Collectively, these results clearly provided evidence for treatment efficacy in the motor strength, coordination and gait parameters at 6 months post injection of AAV1.NT‐3 in the SOD1KO mice by protecting the function comparable to the levels from age‐matched WT mice. We did not observe significant weight difference among UT and NT‐3 treated cohorts and WT mice (data not shown).

### Neurotrophin‐3 gene therapy‐induced histological improvements in SOD1KO muscle and nerve

Histological analyses of hindlimb and forelimb muscles from AAV1.NT‐3 treated and untreated control SOD1KO mice were done at 6 months post gene injection using H&E as routine histopathological stain and succinic dehydrogenase (SDH) and cytochrome C oxidase (COX) enzyme histochemistry stains. Quantitative analysis was carried out on SDH stained sections to determine the metabolic fibre types, namely slow twitch oxidative (STO or type 1), fast twitch oxidative (FTO, or type 2A) and FTG (or type 2B) fibres sampled from deep, intermediate and superficial zones of each muscle as shown previously.^20^

**Figure 2:**
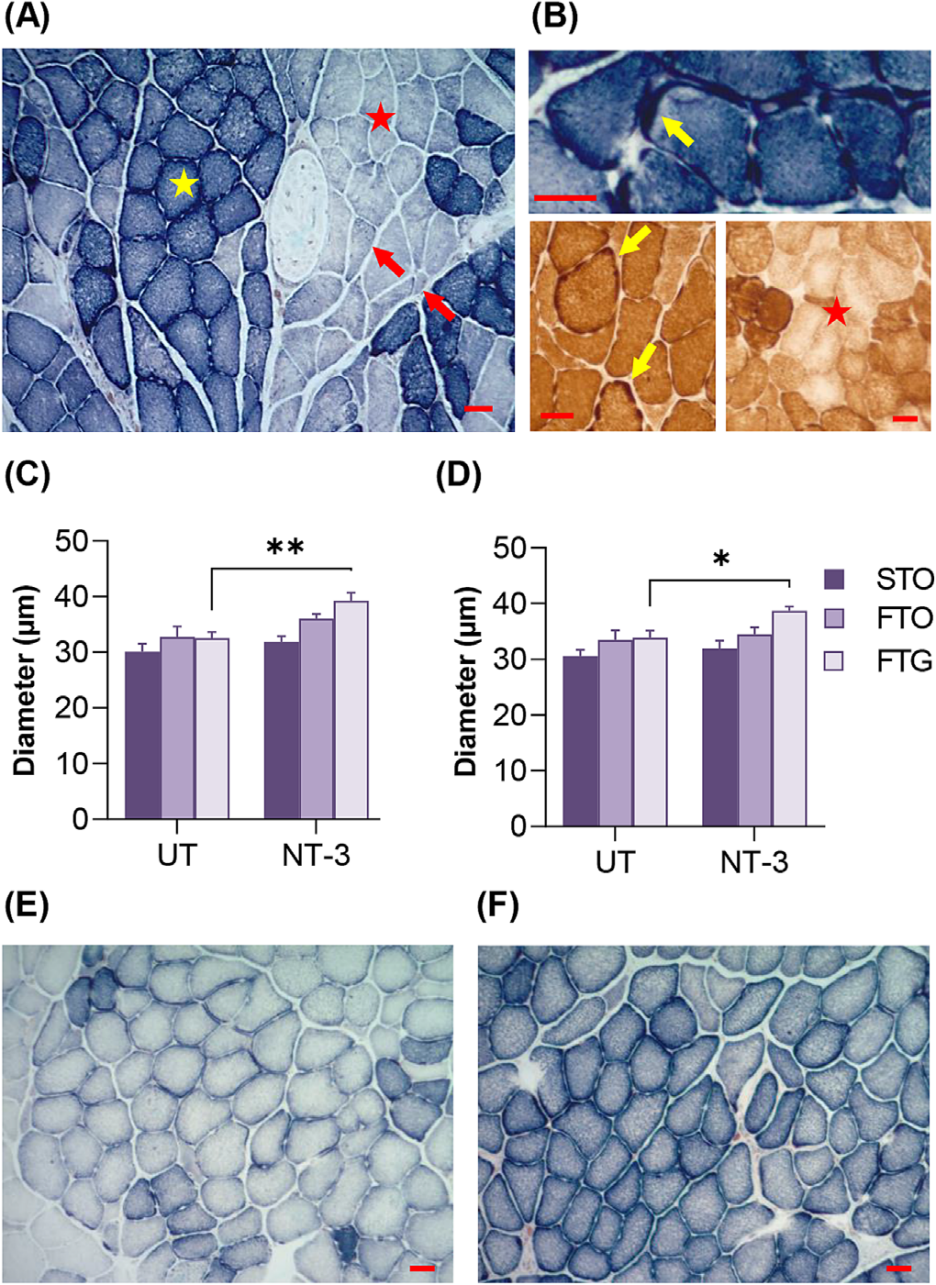
Muscle histochemistry in SOD1KO muscle. (A) Gastrocnemius muscle of an untreated SOD1KO mouse showing muscle fibre size variability, atrophic angular FTG fibres (red arrows) and oxidative (yellow star) and glycolytic (red star) fibre type groupings as neurogenic change with SDH stain. (B) Subsarcolemmal mitochondria accumulation (arrows) depicted as ragged blue (with SDH)/ragged brown (with COX) fibres and COX deficient fibres (star) in muscles from untreated SOD1KO mouse. (C) Bar graph shows increase in fibre diameter in gastrocnemius muscle (*n* = 7 untreated, *n* = 11 treated, *P* = 0.01). (D) Bar graph shows increase in fibre diameter in tibialis anterior muscle (*n* = 6 untreated, *n* = 6 treated, *P* = 0.021). (E, F) Representative images from superficial and deep regions of an SDH‐stained cross section from tibialis anterior muscle in an NT‐3 treated SOD1KO mouse. Scale bar is 30 μm for all images. Error bars are ± SEM, two‐way ANOVA, Bonferroni's multiple comparisons test.

Sarcopenic changes were frequently observed in gastrocnemius and tibialis anterior muscles of the hindlimb; both muscles showed atrophic angular fibres, predominantly belonging to FTG, that is, type 2B fibres, as well as groups of angular atrophied fibres and fibre type groupings as evidence of neurogenic change in some samples (*Figure* 2A). In addition, excessive subsarcolemmal mitochondria content presented as ragged blue or ragged brown fibres (on SDH and COX stains respectively) as well as focal areas of enzyme attenuation or loss were noted (*Figure* 2B). These histopathological changes and decreased muscle mass were more prominent in the gastrocnemius muscle as previously reported.^19^ Compared with aged‐matched WT, the mean muscle fibre diameter of untreated SOD1KO showed 9.4% and 11.4% decrease in tibialis and gastrocnemius muscles respectively and the size decrease was greater in the FTG fibre type of gastrocnemius (21.0% in gastrocnemius vs. 18.5% in tibialis anterior; *Table* S1, S2). The FTG atrophy in this model was more prominent for males in both muscles with diameters about 29% less than WT counterparts. FTG atrophy in female SOD1KO, however, was more prominent in gastrocnemius muscle (23.1% in gastrocnemius vs. 16.7 in tibialis anterior; *Tables* S1 and S2). In addition, compared with WT, a significant fibre type switch to FTG subtype was present in both tibialis anterior and gastrocnemius muscles in SOD1KO without sex difference.

AAV1.NT3 gene therapy resulted in a reversal of muscle atrophy; in fact, in tibialis anterior and gastrocnemius muscles, we observed normalization of the mean fibre size compared with age‐matched WT BL6 controls, as illustrated in fibre‐size distribution histograms, with a shift to left and a significant decrease in small fibre subpopulation, with diameters less than 30 μm (*Figure* S2). Interestingly, in both muscles the fibre size increase was most prominent in the FTG fibre type and in males (*Figure* 2C–F, *Tables* S1 and S2) (tibialis anterior: 23.3% vs. 6.0%; gastrocnemius: 21.3% vs. 18.1% for male and female, respectively). The treatment did not alter fibre type composition of these muscles significantly; however, a modest increase in the percentage of oxidative fibres (STO in tibialis anterior and FTO in gastrocnemius), along with a decrease in the FTG fibres occurred in both muscles of females, suggesting a switch from glycolytic toward oxidative fast twitch type (*Figures* *S3–S4*). As we observed in the naturally occurring sarcopenia model of aged BL6 mice, the triceps muscle from SOD1KO mice showed resistance to sarcopenia to start with, and no treatment effect was noted in fibre size compared with untreated cohort (*Table* S3) although modest but not significant switch to STO in females and FTO in males was noted (*Figure* *S5*).

**Figure 3:**
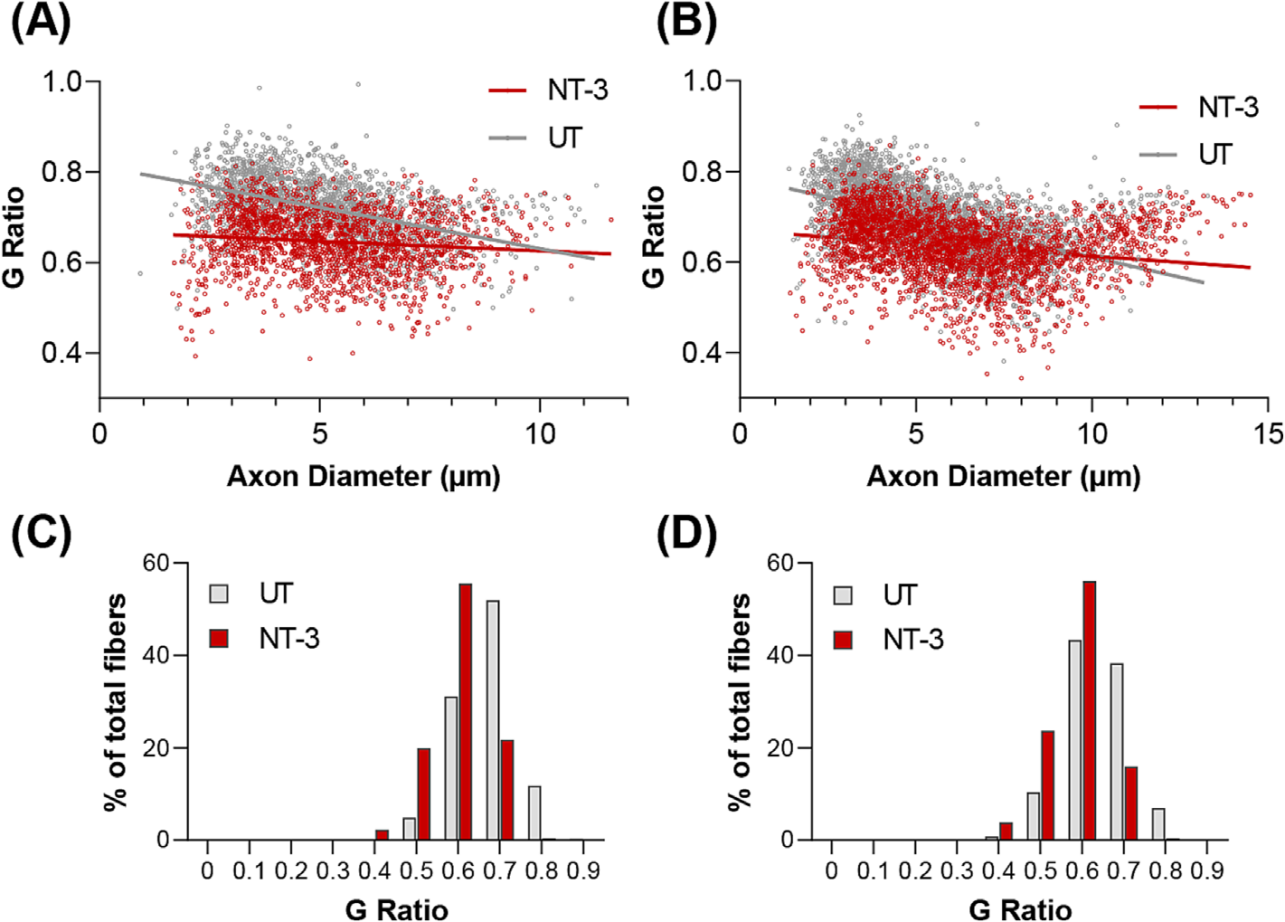
NT‐3 effect on myelin thickness. Scattergrams show that G ratio of both (A) tibial and (B) sciatic nerves were significantly decreased G ratio indicating thicker myelin in treated group compared with the untreated cohort (tibial, *n* = 4 for both cohorts, NT‐3, *r* ^2^ = 0.0127; UT, *r* ^2^ = 0.2014; sciatic, *n* = 6 for both cohorts, NT‐3, *r* ^2^ = 0.0400; UT, *r* ^2^ = 0.2409; linear regression, slopes are significantly different for both nerves analysed, *P* < 0.0001). The shift toward thicker myelin can also be observed in percent distribution graph with treatment in (C) tibial and (D) sciatic nerves.

Examination of the peripheral nerves from 1 year old untreated SOD1KO mice revealed a distally prominent decrease in myelin thickness compared with age‐matched WT mice (*Table* S4). The increase in G ratio, as a measure of thinner myelin, was more prominent in females, although slopes from males and females in linear regression analysis were not significantly different. With NT‐3 gene therapy, we observed a reversal of the hypomyelination state with significantly decreased mean G ratios, toward WT values (*Table* S4), and scatter plots having significantly different slopes between treated and untreated groups (*Figure* 3A,B), as well as with a shift toward thicker myelin, in per cent G ratio distribution graphs (*Figure* 3C,D).

### Neurotrophin‐3 gene therapy‐induced alterations in mitochondrial and glycolysis markers and mTORC1 activation in SOD1KO muscle

**Figure 4:**
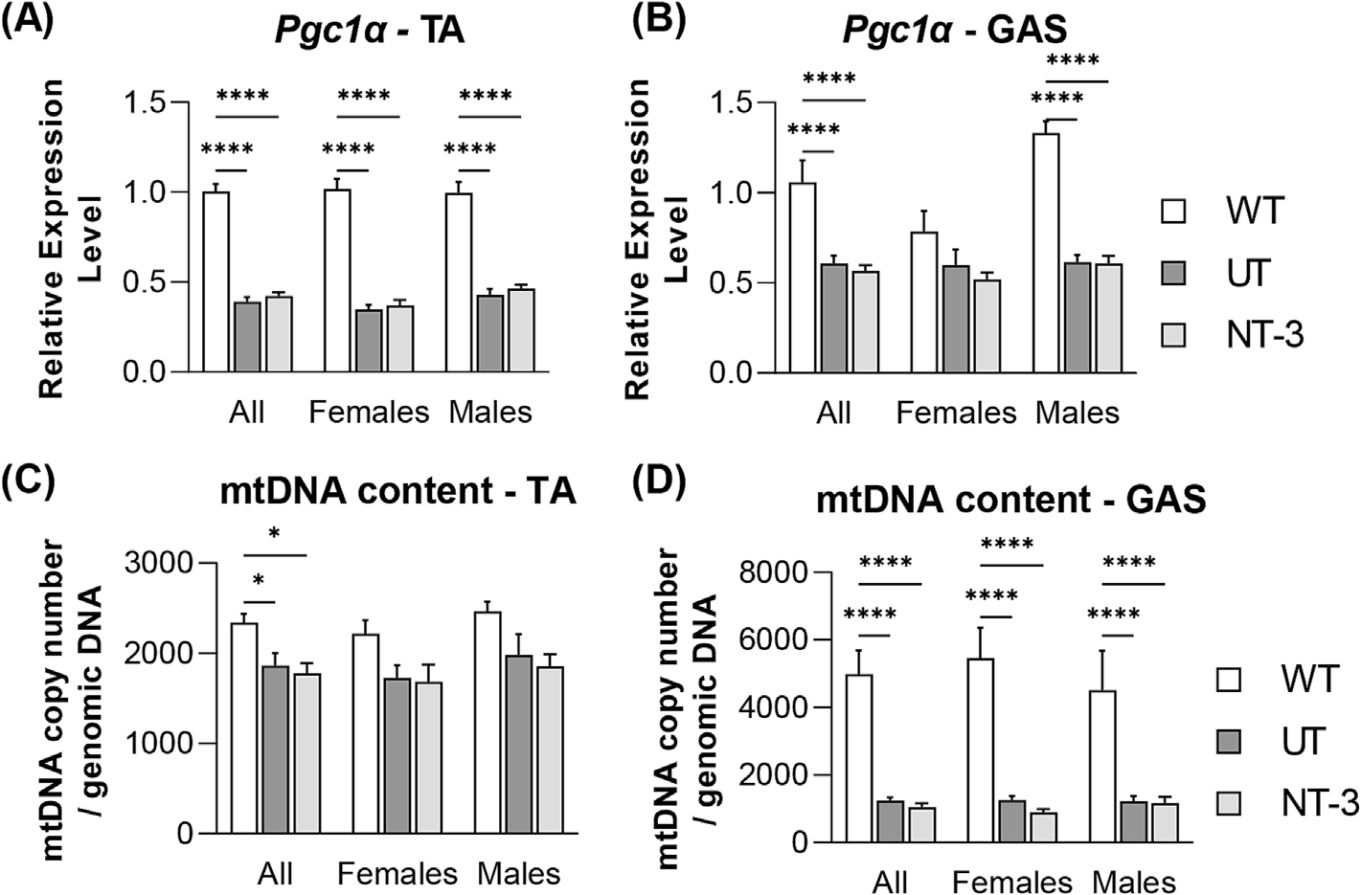
Mitochondrial biogenesis in SOD1KO model. Bar graphs represent relative expression levels of (A, B) *Pgc1α* and (C, D) mitochondrial DNA copy number/genomic DNA in (A, C) tibialis anterior and *(B, D)* gastrocnemius muscle from treated and untreated SOD1KO mice along with age‐matched wilt type mice. Data are represented as mean ± SEM; *P < 0.05, ****P < 0.0001, two‐way ANOVA, Tukey's multiple comparisons test. *N* = 11, NT‐3; *n* = 13, UT; *n* = 8, WT; even sex‐distribution for all cohorts.

**Figure 5:**
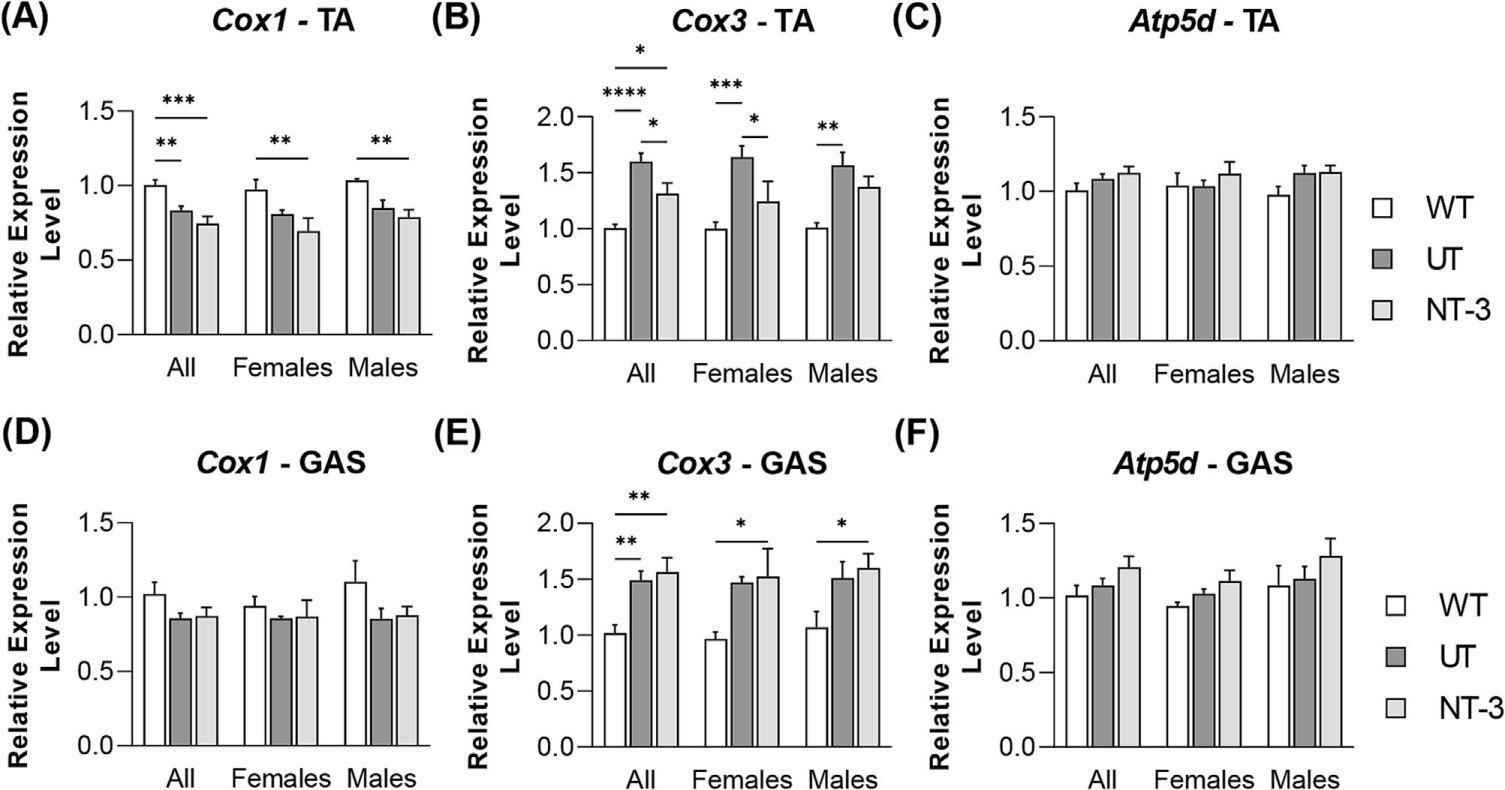
Expression levels of mitochondrial proteins. Bar graphs represent relative expression levels of (A, D) Cox1, (B, E) Cox3 and (C, F) Atp5d in (A–C) tibialis anterior and (D–F) gastrocnemius muscle from treated and untreated SOD1KO mice along with age‐matched wilt type mice. *N* = 11, NT‐3; *n* = 13, UT; *n* = 8, WT; even sex‐distribution for all cohorts. Data are represented as mean ± SEM; *P < 0.05, **P < 0.01, ***P < 0.001, ****P < 0.0001, two‐way ANOVA, Tukey's multiple comparisons test.

As expected in SOD1KO sarcopenic muscle, expression levels of *pgc1α* and mitochondria copy numbers were significantly reduced in tibialis anterior and gastrocnemius muscles from the untreated SOD1KO compared with age‐matched WT (*Figure* 4A–D). In addition, a decrease in *cox1* transcripts along with an increase in *cox3* was present while *atp5d* transcripts did not change in both muscle from both sexes (*Figure* 5A–F). These findings indicate defective mitochondria biogenesis and mitochondria loss, compatible with change in the composition of fibre type, leading to a reduction in the oxidative fibres in the untreated SOD1KO muscle at 1 year of age. Overall, the treatment had no significant effect on these transcripts with no sex difference, except in the tibialis anterior muscle, where there was a reversal of, what appears to be, a compensatory increase in *Cox3* expression (*Figure* 5B).

**Figure 6:**
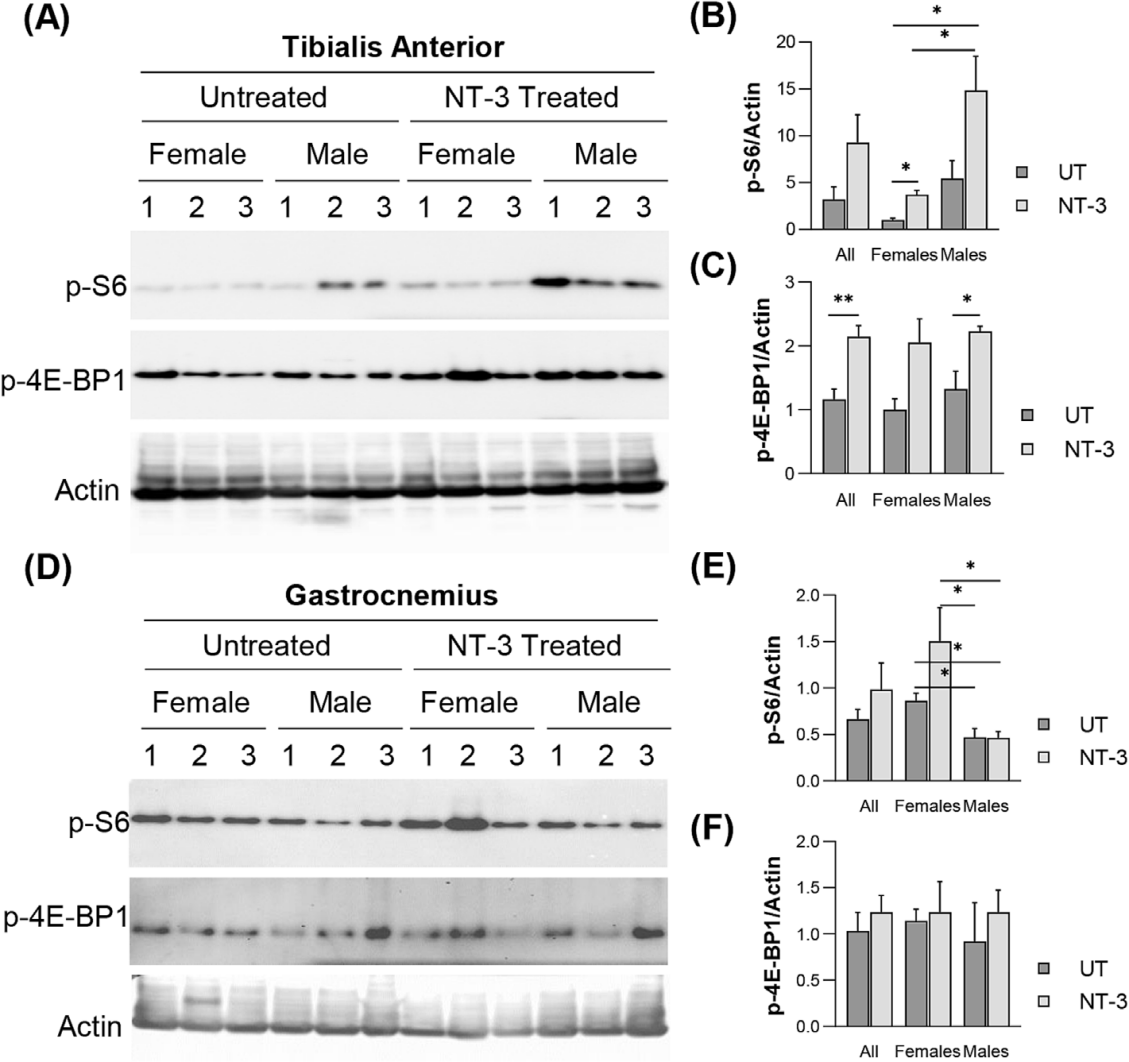
WB –Western blots showing the expression level of p‐S6, and p‐4E‐BP1 proteins in (A) tibialis anterior and (D) gastrocnemius muscles. Protein levels of (B, E) p‐S6 and (E, F) p‐4E‐BP1 normalized to actin (*n* = 6 for both cohorts with equal sex distribution). Data are represented as mean ± SEM; *P < 0.05, **P < 0.01, unpaired *t*‐test.

**Figure 7:**
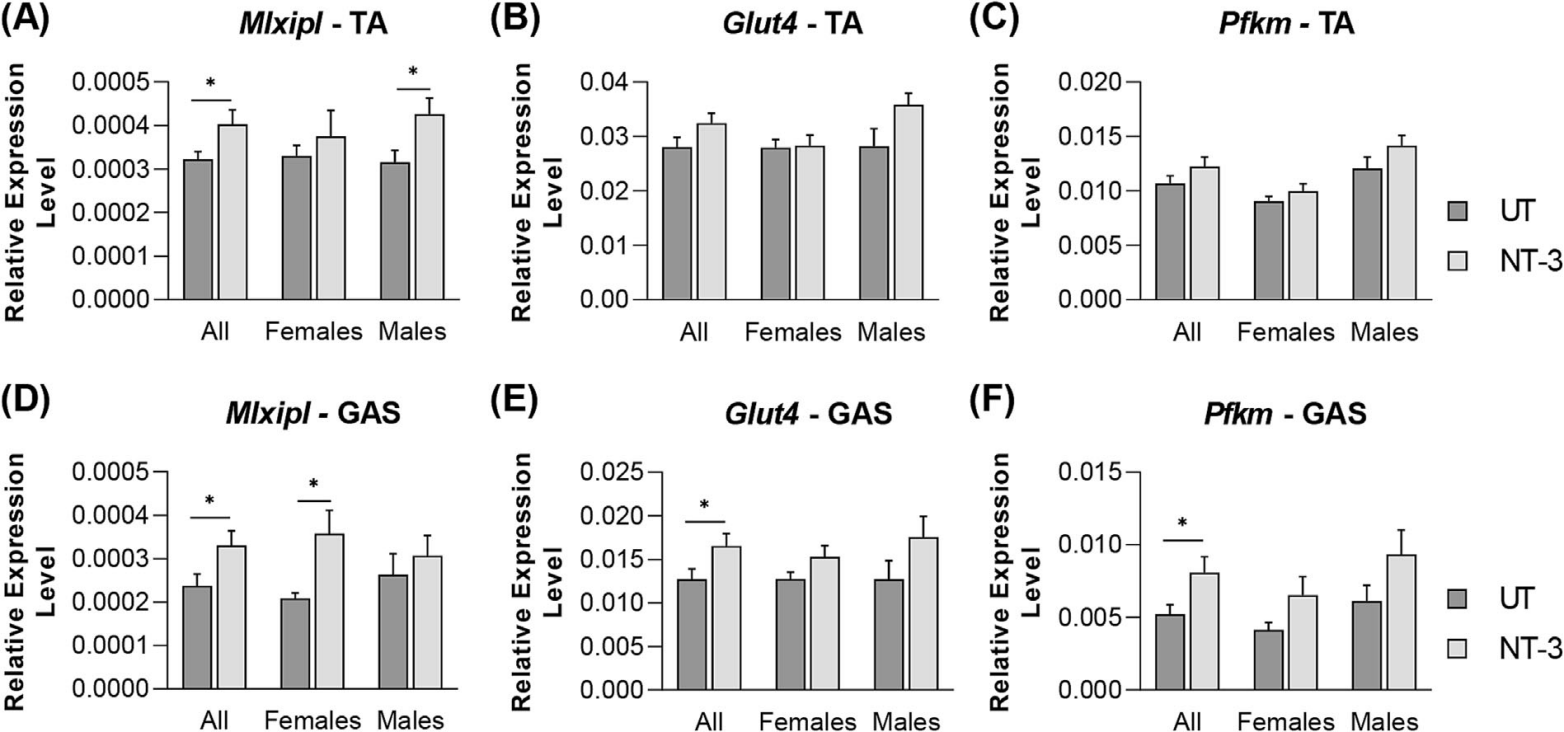
Expression levels of glycolytic pathway markers. Bar graphs represent relative expression levels of (A, D) *Mlxipl*, (B, E) *Glut4* and (C, F) *Pfkm* in (A–C) tibialis anterior and (D–F) gastrocnemius muscle from treated and untreated SOD1KO. *N* = 11, NT‐3; *n* = 13, UT; even sex‐distribution for both cohorts. Data are represented as mean ± SEM; *P < 0.05, unpaired *t*‐test.

**Figure 8:**
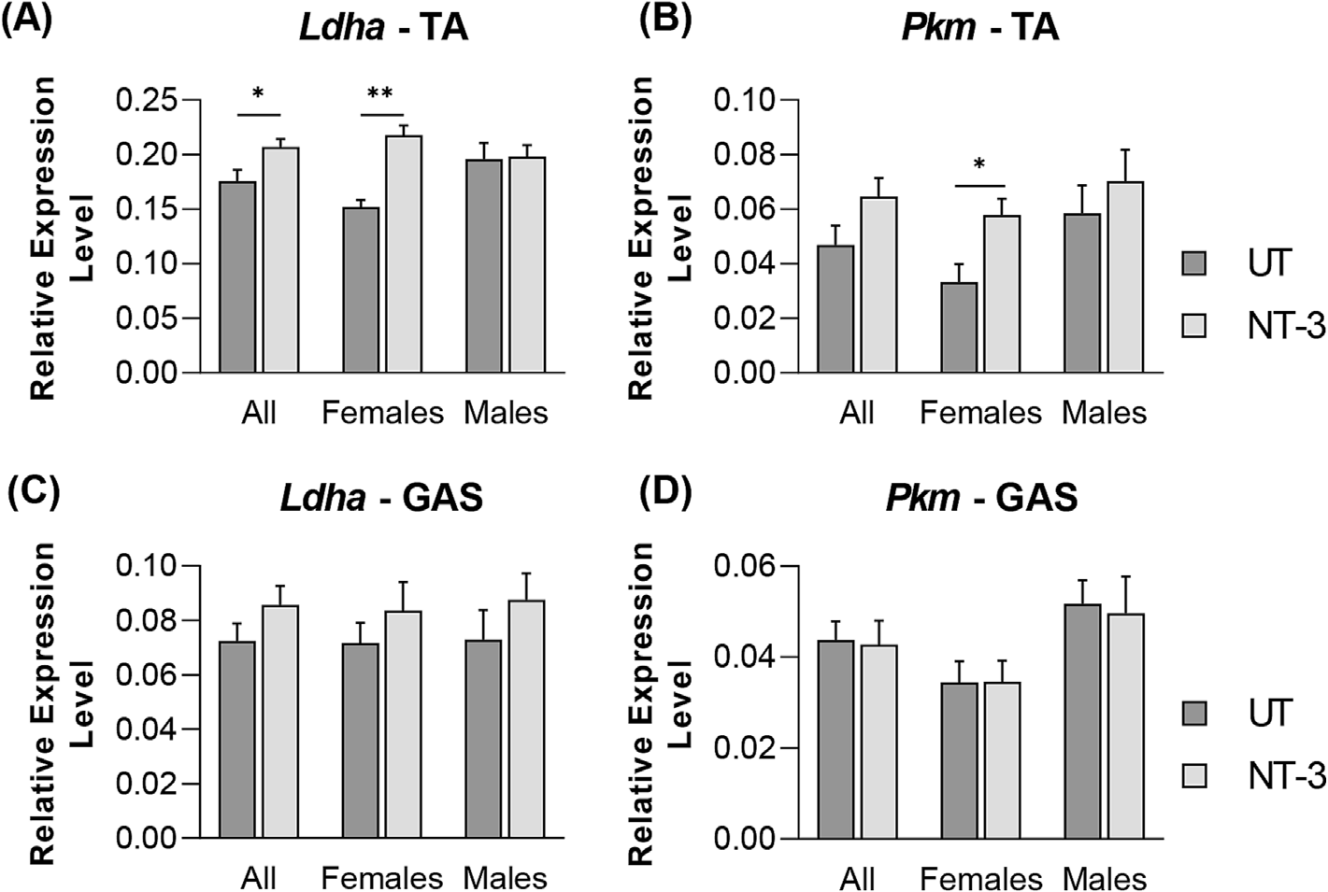
Expression levels of glycolytic pathway markers. Bar graphs represent relative expression levels of (A, C) *Ldha*, and (B, D) *Pkm* in (A, B) tibialis anterior and (C, D) gastrocnemius muscle from treated and untreated SOD1KO. *N* = 11, NT‐3; *n* = 13, UT; even sex‐distribution for both cohorts. Data are represented as mean ± SEM; *P < 0.05, unpaired *t*‐test.

In response to therapy, the presence of an overall fibre size increase, particularly of the glycolytic subtype suggested activation of mTORC1 via NT‐3, as we reported previously.^13^ The activity of mTORC1 was assessed by phosphorylation levels of its substrates, the downstream eukaryotic translation initiation factor 4E (eIF4E)‐binding protein1(4E‐BP1) and ribosomal proteinS6 kinase (S6). Indeed, in WB analysis we found significantly increased p‐S6 and p‐4EBP1 in both sexes, prominent in tibialis anterior from males (*Figure* 6A–C) correlating with significantly increased FTG fibre size in quantitative muscle histology (T*able* S1). The gastrocnemius muscle, however, showed no significant change in p‐S6 and p‐4EBP1 with treatment, although p‐S6 levels were significantly higher in the untreated and treated females, compared with male counterparts (*Figure* 6D–F). To further explore the anabolic effect of NT‐3 on muscle fibre type, we investigated expression levels of the glycolytic pathway markers^24^ in both muscles from the treated and untreated SOD1KO mice (*Figure* 7A–F and *Figure* 8A–D). *MLXipl* (also known as *ChREBP*), the carbohydrate responsive element‐binding protein expression^25^ was increased in both muscle with treatment. This increase showed a sex and muscle specific difference, being significant for males in tibialis anterior (*Figure* 7A), but for females in gastrocnemius (*Figure* 7D). We also investigated the expression level of *Glut4*, which is an insulin‐regulated glucose transporter predominantly found in muscles cells, thus critical in regulating glucose uptake and metabolism in muscles.^26^ We found an increased trend for this marker in both muscles for males, especially in gastrocnemius muscle. Next, we investigated PFKm, muscle phosphofructokinase‐1, which is one of the most important regulatory enzymes of glycolysis. *Pfkm* also showed a similar increase trend in males as *Glut4*. *Ldha*, the M isoform of LDH (expressed in skeletal muscle), involved in glycogen final stage breakdown,^27^ and *Pkm*, involved in final stage of glycolysis for pyruvate production, were significantly increased in female tibialis anterior, while no significant change was detected in gastrocnemius muscle (*Figure* 8A–D). Collectively, these findings show that NT‐3 induced remodelling of carbohydrate metabolism, upregulating glucose uptake and glycolytic pathways in muscle for energy source and that this anabolic effect is manifested in FTG fibres as increased diameter predominantly in the tibialis anterior muscle. In contrast, gastrocnemius muscle response to NT‐3 appears to be much subdued, particularly in males compared with females.

## Discussion

In this study, we present evidence for the efficacy of AAV1.NT‐3 gene therapy in the SOD1KO mouse, a model for accelerated aging. AAV1.NT‐3 was introduced to this model via IM injection when mice were at 6 months of age, which led to significant improvements in the functional, histological, and molecular parameters, as documented with midpoint and endpoint studies at the age of 9 and 12 months. The endpoint circulating NT‐3 levels were detected only in the treated mice, as we reported previously in other disease models.^14^, ^15^, ^16^ We also found that exercise capacity, endurance, and motor coordination of the mice assessed by treadmill and rotarod tests were preserved throughout study in the NT‐3 treated cohort. Moreover, the quantitative histopathologic assessment of muscles provided additional evidence in support of the functional outcome with treatment. Compatible with the previously reported muscle mass loss with aging in the SOD1KO mouse,^19^ we found fibre size decrease in the distal limb muscles most prominent in the gastrocnemius for FTG (type 2B) fibres^9^ in the untreated cohort and determined that NT‐3 treatment stabilized the fibre diameter within the values similar to those observed in the age‐matched WT counterparts. Interestingly, the triceps muscle appeared unaffected by aging in this model as we observed in the naturally aged C57Bl6 mice. We found no fibre size alterations in the triceps muscle from one‐year‐old untreated SOD1KO mice and observed no significant fibre size change of this muscle with NT‐3 gene therapy as well compared with WT counterparts. These observations in triceps muscle is consistent with our previous findings suggesting that NT‐3 does not exert its effects on well‐differentiated or normal functioning cells, but rather is operative upon remodelled‐cell metabolism that may result from a pathological process.^13^, ^28^ Contrasting with this, NT‐3 effect as an increase of myelin thickness was present in both sciatic and tibial nerves from this model, in which we showed the presence of a hypomyelination state as part of the phenotype to begin with.

Previous publications demonstrated that in the SOD1KO mice, there is a significant increase of oxidative stress in muscle cells causing mitochondria dysfunction, and thus accelerating muscle atrophy and NMJ disruption.^6^, ^9^, ^10^ Mitochondria are the major endogenous source of superoxide^29^; therefore, the functional status of mitochondria and its biogenesis‐related genes are of great importance for understanding the efficacy of NT‐3. To explore this, we first investigated the expression levels of mitochondria biogenesis marker pgc1α and functional markers *atp5d*, *Cox1*, *Cox3* and mitochondria DNA content in tibialis anterior and gastrocnemius muscle. Our results showed that there was little change in expression of these markers and the mitochondria copy number between untreated and NT‐3 treated SOD1KO mice. On the other hand, the quantitative histologic data demonstrating significantly increased fibre diameter of FTG in both tibialis anterior and gastrocnemius muscle strongly suggested that NT‐3 may affect the glucose uptake and metabolic pathways in these muscles. This premise was investigated with additional studies, which showed an overall increase in expression levels of *Mlxipl*, *Glut4*, *Pfkm*, *Ldha* and *Pkm* in both muscles and indicating that glycolytic pathway in these muscles have been upregulated with NT‐3 treatment.

Taken together, our data indicate that NT‐3 facilitates FTG function by upregulating glucose uptake and glycolytic pathways, rather than improving mitochondria function in the SOD1KO muscle. This is further supported by findings in the tibialis anterior muscle that the phosphorylation level of S6 was significantly increased after NT‐3 treatment in both male and female SOD1KO mice. In addition, the phosphorylation of 4EBP1 also showed a trend of increase after NT‐3 treatment. These observations confirm our previous findings that NT‐3 has the potential to activate the mTORC pathway.^13^, ^30^ mTOR, is a central regulator of cellular growth and metabolism, and the mTORC pathway is known to sense and integrate a variety of environmental cues to regulate muscle mass and coordinates protein and lipid synthesis, oxidative metabolism, and glucose homeostasis, activated in response to growth factors via the PI3K/Akt pathway.^31^, ^32^ It is also widely recognized for maintaining muscle mass and fibre size, particularly of glycolytic fibres. It has been shown that the low oxidative muscle fibres have a larger potential to phosphorylate S6 kinase1 compared with high oxidative fibres, in relation to increases in translation initiation and subsequent gain in muscle mass.^33^, ^34^, ^35^

We want to emphasize the similarities and differences in response to NT‐3 gene therapy between the accelerated sarcopenia model resulting from underlying increased oxidative stress/mitochondria dysfunction in SOD1KO mice at 1 year of age and the naturally occurring sarcopenia model, Bl6 mice at 2 years of age. Prominent atrophy of FTG fibres as hallmark of sarcopenia, was noted to be more severe in the SOD1KO males than aged Bl6 males. Comparatively, at 6 months post gene delivery, the mean muscle fibre size of tibialis anterior was normalized toward 1‐year‐old WT values in both models with prominent increase of FTG fibre size. The difference however was the potential of NT‐3 to increase *Atp5d* expression levels and mitochondria content along with increased COX enzyme stain intensity, was more prominent in females, which we believe is due to response capacity of the muscle to mTOR activation in the natural aging model. In the female tibialis anterior, we found significant increases in the downstream targets of mTORC1, p‐S6 kinase and p‐4E‐BP1 proteins, indicating activation of mTORC1 which correlated with increased radial growth of FTG fibres.^17^ Via 4E‐BPs, mTORC1 regulates synthesis of nucleus‐encoded mitochondrial proteins (such as Atp5d), controls mitochondrial activity and biogenesis, and therefore, coordinates energy consumption and production,^36^ which is reflected as increased radial growth of oxidative fibres. In contrast, the current data from the SOD1KO muscle indicates that NT‐3 facilitates only the FTG function by upregulating glucose uptake and glycolytic pathways, and not having a significant effect on mitochondria function, likely due to existing oxidative stress and damage to mitochondria.^6^ In conclusion, our findings indicate that AAV1.NT‐3 gene therapy has beneficial protective effects for the accelerated aging/sarcopenia mouse model, SOD1KO. We show that NT‐3, through the activation of mTOR and glycolytic pathways maintained FTG fibre size and functional performance in this model and normalized the myelin thickness as well.

## Conflict of interest statement

The authors declare that there is no conflict of interest.

## Supporting information


**Figure S1.** scAAV1.tMCK.NT‐3 vector and serum NT‐3 levels. (A) NT‐3 expression is driven by a tMCK in the scAAV vector backbone. The diagram shows the cassette composed of a tMCK enhancer/promoter region (714 bp), the full‐length NT‐3 cDNA (774 bp) and the SV40 polyA tail (211 bp); Ref#15. (B) Serum samples were collected from treated (NT‐3) and untreated (UT) mice, and NT‐3 levels were measured by ELISA. Error bars are ± SEM; *n* = 5 for treated cohort, *n* = 4 for untreated cohort. NT‐3 serum levels were below detection range for untreated mice.
Figure S2. Fibre size distribution in treated and untreated SOD1KO mice and age‐matched wild‐type mice. (A) Tibialis anterior fibre size distribution (*n* = 6, NT‐3; n = 6, UT; *n* = 4, WT. (B) Gastrocnemius fibre size distribution (*n* = 11, NT‐3; *n* = 7, UT; n = 4, WT). Data are represented as mean ± SEM; *P.
Figure S3. Graphs represent the changes in the percent distribution of (A) STO, (B) FTO and (C) FTG fibre types in the tibialis anterior muscle from UT and treated cohorts and agematched WT mice, shown as sexes combined (black) and separated (blue for males, red for females). *n* = 6, NT‐3; n = 6, UT; *n* = 8, WT; even sex‐distribution for all cohorts. Data are represented as mean ± SEM.
Figure S4. Graphs represent the changes in the percent distribution of (A) STO, (B) FTO and (C) FTG fibre types in the gastrocnemius muscle from UT and treated cohorts and agematched WT mice, shown as sexes combined (black) and separated (blue for males, red for females). *n* = 11, NT‐3; *n* = 7, UT; *n* = 8, WT; even sex‐distribution for all cohorts. Data are represented as mean ± SEM.
Figure S5. Graphs represent the changes in the percent distribution of (A) STO, (B) FTO and (C) FTG fibre types in the triceps muscle from UT and treated cohorts and age‐matched WT mice, shown as sexes combined (black) and separated (blue for males, red for females). *n* = 6, NT‐3; n = 6, UT; *n* = 8, WT; even sex‐distribution for all cohorts. Data are represented as mean ± SEM.
Table S1 Fibre size analysis on tibialis anterior muscle of NT‐3 treated and untreated SOD1KO mice.
Table S2 Fibre size analysis on gastrocnemius muscle of NT‐3 treated and untreated SOD1KO mice.
Table S3 Fibre size analysis on triceps muscle of NT‐3 treated and untreated SOD1KO mice.
Table S4 Average G ratios of the tibial and sciatic nerves from SOD1KO mice.

## References

[1] Roubenoff R . Sarcopenia and its implications for the elderly. Eur J Clin Nutr 2000;54:S40–S47. 11041074 10.1038/sj.ejcn.1601024

[2] Mansouri A , Muller FL , Liu Y , Ng R , Faulkner J , Hamilton M , et al. Alterations in mitochondrial function, hydrogen peroxide release and oxidative damage in mouse hind‐limb skeletal muscle during aging. Mech Ageing Dev 2006;127:298–306. 16405961 10.1016/j.mad.2005.11.004

[3] Moylan JS , Reid MB . Oxidative stress, chronic disease, and muscle wasting. Muscle Nerve 2007;35:411–429. 17266144 10.1002/mus.20743

[4] Choksi KB , Nuss JE , Deford JH , Papaconstantinou J . Age‐related alterations in oxidatively damaged proteins of mouse skeletal muscle mitochondrial electron transport chain complexes. Free Radic Biol Med 2008;45:826–838. 18598756 10.1016/j.freeradbiomed.2008.06.006 PMC2873767

[5] Figueiredo PA , Powers SK , Ferreira RM , Appell HJ , Duarte JA . Aging impairs skeletal muscle mitochondrial bioenergetic function. J Gerontol A Biol Sci Med Sci 2009;64:21–33. 19196905 10.1093/gerona/gln048 PMC2691197

[6] Muller FL , Song W , Liu Y , Chaudhuri A , Pieke‐Dahl S , Strong R , et al. Absence of CuZn superoxide dismutase leads to elevated oxidative stress and acceleration of age‐dependent skeletal muscle atrophy. Free Radic Biol Med 2006;40:1993–2004. 16716900 10.1016/j.freeradbiomed.2006.01.036

[7] Perez VI , Bokov A , Van Remmen H , Mele J , Ran Q , Ikeno Y , et al. Is the oxidative stress theory of aging dead? Biochim Biophys Acta 2009;1790:1005–1014. 19524016 10.1016/j.bbagen.2009.06.003 PMC2789432

[8] Xie WQ , He M , Yu DJ , Wu YX , Wang XH , Lv S , et al. Mouse models of sarcopenia: classification and evaluation. J Cachexia Sarcopenia Muscle 2021;12:538–554. 33951340 10.1002/jcsm.12709 PMC8200444

[9] Jang YC , Lustgarten MS , Liu Y , Muller FL , Bhattacharya A , Liang H , et al. Increased superoxide in vivo accelerates age‐associated muscle atrophy through mitochondrial dysfunction and neuromuscular junction degeneration. FASEB J 2010;24:1376–1390. 20040516 10.1096/fj.09-146308 PMC2987499

[10] Deepa SS , van Remmen H , Brooks SV , Faulkner JA , Larkin L , McArdle A , et al. Accelerated sarcopenia in Cu/Zn superoxide dismutase knockout mice. Free Radic Biol Med 2019;132:19–23. 30670156 10.1016/j.freeradbiomed.2018.06.032 PMC6405207

[11] Fischer LR , Li Y , Asress SA , Jones DP , Glass JD . Absence of SOD1 leads to oxidative stress in peripheral nerve and causes a progressive distal motor axonopathy. Exp Neurol 2012;233:163–171. 21963651 10.1016/j.expneurol.2011.09.020 PMC4068963

[12] Fischer LR , Igoudjil A , Magrane J , Li Y , Hansen JM , Manfredi G , et al. SOD1 targeted to the mitochondrial intermembrane space prevents motor neuropathy in the Sod1 knockout mouse. Brain 2011;134:196–209. 21078595 10.1093/brain/awq314 PMC3009841

[13] Yalvac ME , Amornvit J , Chen L , Shontz KM , Lewis S , Sahenk Z . AAV1.NT‐3 gene therapy increases muscle fiber diameter through activation of mTOR pathway and metabolic remodeling in a CMT mouse model. Gene Ther 2018;25:129–138. 29523879 10.1038/s41434-018-0009-8

[14] Sahenk Z , Galloway G , Clark KR , Malik V , Rodino‐Klapac LR , Kaspar BK , et al. AAV1.NT‐3 gene therapy for charcot‐marie‐tooth neuropathy. Mol Ther 2014;22:511–521. 24162799 10.1038/mt.2013.250 PMC3944324

[15] Ozes B , Myers M , Moss K , McKinney J , Ridgley A , Chen L , et al. AAV1.NT‐3 gene therapy for X‐linked Charcot‐Marie‐Tooth neuropathy type 1. Gene Ther 2021;29:127–137. 33542455 10.1038/s41434-021-00231-3 PMC9013664

[16] Ozes B , Moss K , Myers M , Ridgley A , Chen L , Murrey D , et al. AAV1.NT‐3 gene therapy in a CMT2D model: phenotypic improvements in Gars(P278KY/+) mice. Brain Commun 2021;3:fcab252. 34755111 10.1093/braincomms/fcab252 PMC8568849

[17] Ozes B , Tong L , Myers M , Moss K , Ridgley A , Sahenk Z . AAV1NT‐3 gene therapy prevents age‐related sarcopenia. Aging (Albany NY) 2023;15:1306–1329. 36897179 10.18632/aging.204577 PMC10042697

[18] Flood DG , Reaume AG , Gruner JA , Hoffman EK , Hirsch JD , Lin YG , et al. Hindlimb motor neurons require Cu/Zn superoxide dismutase for maintenance of neuromuscular junctions. Am J Pathol 1999;155:663–672. 10433959 10.1016/S0002-9440(10)65162-0 PMC1866863

[19] Deepa SS , Bhaskaran S , Espinoza S , Brooks SV , McArdle A , Jackson MJ , et al. A new mouse model of frailty: the Cu/Zn superoxide dismutase knockout mouse. GeroScience 2017;39:187–198. 28409332 10.1007/s11357-017-9975-9 PMC5411367

[20] Sahenk Z , Ozes B , Murrey D , Myers M , Moss K , Yalvac ME , et al. Systemic delivery of AAVrh74.tMCK.hCAPN3 rescues the phenotype in a mouse model for LGMD2A/R1. Mol Ther Methods Clin Dev 2021;22:401–414. 34514031 10.1016/j.omtm.2021.06.010 PMC8413669

[21] Wang L , Xu X , Jiang C , Ma G , Huang Y , Zhang H , et al. mTORC1‐PGC1 axis regulates mitochondrial remodeling during reprogramming. FEBS J 2020;287:108–121. 31361392 10.1111/febs.15024

[22] Chaignat E , Yahya‐Graison EA , Henrichsen CN , Chrast J , Schutz F , Pradervand S , et al. Copy number variation modifies expression time courses. Genome Res 2011;21:106–113. 21084671 10.1101/gr.112748.110 PMC3012917

[23] Jousse C , Muranishi Y , Parry L , Montaurier C , Even P , Launay JM , et al. Perinatal protein malnutrition affects mitochondrial function in adult and results in a resistance to high fat diet‐induced obesity. PLoS ONE 2014;9:e104896. 25118945 10.1371/journal.pone.0104896 PMC4132016

[24] Mattos SEC , Diel LF , Bittencourt LS , Schnorr CE , Goncalves FA , Bernardi L , et al. Glycolytic pathway candidate markers in the prognosis of oral squamous cell carcinoma: a systematic review with meta‐analysis. Braz J Med Biol Res 2021;54:e10504. 33503201 10.1590/1414-431X202010504 PMC7836401

[25] Hunt LC , Xu B , Finkelstein D , Fan Y , Carroll PA , Cheng PF , et al. The glucose‐sensing transcription factor MLX promotes myogenesis via myokine signaling. Genes Dev 2015;29:2475–2489. 26584623 10.1101/gad.267419.115 PMC4691951

[26] Thiel G , Guethlein LA , Rossler OG . Insulin‐responsive transcription factors. Biomolecules 2021;11:11. 10.3390/biom11121886 PMC8699568 34944530

[27] Klein R , Nagy O , Tothova C , Chovanova F . Clinical and diagnostic significance of lactate dehydrogenase and its isoenzymes in animals. Vet Med Int 2020;2020:5346483. 32607139 10.1155/2020/5346483 PMC7313120

[28] Young C , Miller E , Nicklous DM , Hoffman JR . Nerve growth factor and neurotrophin‐3 affect functional recovery following peripheral nerve injury differently. Restor Neurol Neurosci 2001;18:167–175. 11847440

[29] Green DR , Amarante‐Mendes GP . The point of no return: mitochondria, caspases, and the commitment to cell death. Results Probl Cell Differ 1998;24:45–61. 9949831 10.1007/978-3-540-69185-3_3

[30] Coelho RP , Yuelling LM , Fuss B , Sato‐Bigbee C . Neurotrophin‐3 targets the translational initiation machinery in oligodendrocytes. Glia 2009;57:1754–1764. 19455580 10.1002/glia.20888 PMC4300950

[31] Morita M , Gravel SP , Hulea L , Larsson O , Pollak M , St‐Pierre J , et al. mTOR coordinates protein synthesis, mitochondrial activity and proliferation. Cell Cycle 2015;14:473–480. 25590164 10.4161/15384101.2014.991572 PMC4615141

[32] Laplante M , Sabatini DM . mTOR signaling in growth control and disease. Cell 2012;149:274–293. 22500797 10.1016/j.cell.2012.03.017 PMC3331679

[33] Baar K , Esser K . Phosphorylation of p70(S6k) correlates with increased skeletal muscle mass following resistance exercise. Am J Physiol 1999;276:C120–C127. 9886927 10.1152/ajpcell.1999.276.1.C120

[34] Koopman R , Zorenc AH , Gransier RJ , Cameron‐Smith D , van Loon LJ . Increase in S6K1 phosphorylation in human skeletal muscle following resistance exercise occurs mainly in type II muscle fibers. Am J Physiol Endocrinol Metab 2006;290:E1245–E1252. 16434552 10.1152/ajpendo.00530.2005

[35] Hornberger TA , Hunter RB , Kandarian SC , Esser KA . Regulation of translation factors during hindlimb unloading and denervation of skeletal muscle in rats. Am J Physiol 2001;281:C179–C187. 10.1152/ajpcell.2001.281.1.C179 11401840

[36] Morita M , Gravel SP , Chenard V , Sikstrom K , Zheng L , Alain T , et al. mTORC1 controls mitochondrial activity and biogenesis through 4E‐BP‐dependent translational regulation. Cell Metab 2013;18:698–711. 24206664 10.1016/j.cmet.2013.10.001

[37] von Haehling S , Morley JE , Coats AJS , Anker SD . Ethical guidelines for publishing in the Journal of Cachexia, Sarcopenia and Muscle: update 2021. J Cachexia Sarcopenia Muscle 2021;12:2259–2261. 34904399 10.1002/jcsm.12899 PMC8718061

