# Supplementary material for: AAV1.NT‐3 gene therapy in the SOD1KO mouse model of accelerated sarcopenia
Source: J Cachexia Sarcopenia Muscle. 2023 Aug 8;14(5):2204–15. doi: 10.1002/jcsm.13303 (PMC10570084; doi:10.1002/jcsm.13303)
Supplement: Supplementary file 1 — Figure S1. scAAV1.tMCK.NT‐3 vector and serum NT‐3 levels. (A) NT‐3 expression is driven by a tMCK in the scAAV vector backbone. The diagram shows the cassette composed of a tMCK enhancer/promoter region (714 bp), the full‐length NT‐3 cDNA (774 bp) and the SV40 polyA tail (211 bp); Ref#15. (B) Serum samples were collected from treated (NT‐3) and untreated (UT) mice, and NT‐3 levels were measured by ELISA. Error bars are ± SEM; n = 5 for treated cohort, n = 4 for untreated cohort. NT‐3 serum levels were below detection range for untreated mice. Figure S2. Fibre size distribution in treated and untreated SOD1KO mice and age‐matched wild‐type mice. (A) Tibialis anterior fibre size distribution (n = 6, NT‐3; n = 6, UT; n = 4, WT. (B) Gastrocnemius fibre size distribution (n = 11, NT‐3; n = 7, UT; n = 4, WT). Data are represented as mean ± SEM; *P. Figure S3. Graphs represent the changes in the percent distribution of (A) STO, (B) FTO and (C) FTG fibre types in the tibialis anterior muscle from UT and treated cohorts and agematched WT mice, shown as sexes combined (black) and separated (blue for males, red for females). n = 6, NT‐3; n = 6, UT; n = 8, WT; even sex‐distribution for all cohorts. Data are represented as mean ± SEM. Figure S4. Graphs represent the changes in the percent distribution of (A) STO, (B) FTO and (C) FTG fibre types in the gastrocnemius muscle from UT and treated cohorts and agematched WT mice, shown as sexes combined (black) and separated (blue for males, red for females). n = 11, NT‐3; n = 7, UT; n = 8, WT; even sex‐distribution for all cohorts. Data are represented as mean ± SEM. Figure S5. Graphs represent the changes in the percent distribution of (A) STO, (B) FTO and (C) FTG fibre types in the triceps muscle from UT and treated cohorts and age‐matched WT mice, shown as sexes combined (black) and separated (blue for males, red for females). n = 6, NT‐3; n = 6, UT; n = 8, WT; even sex‐distribution for all cohorts. Data are represented as mean ± S [file JCSM-14-2204-s001.pdf]

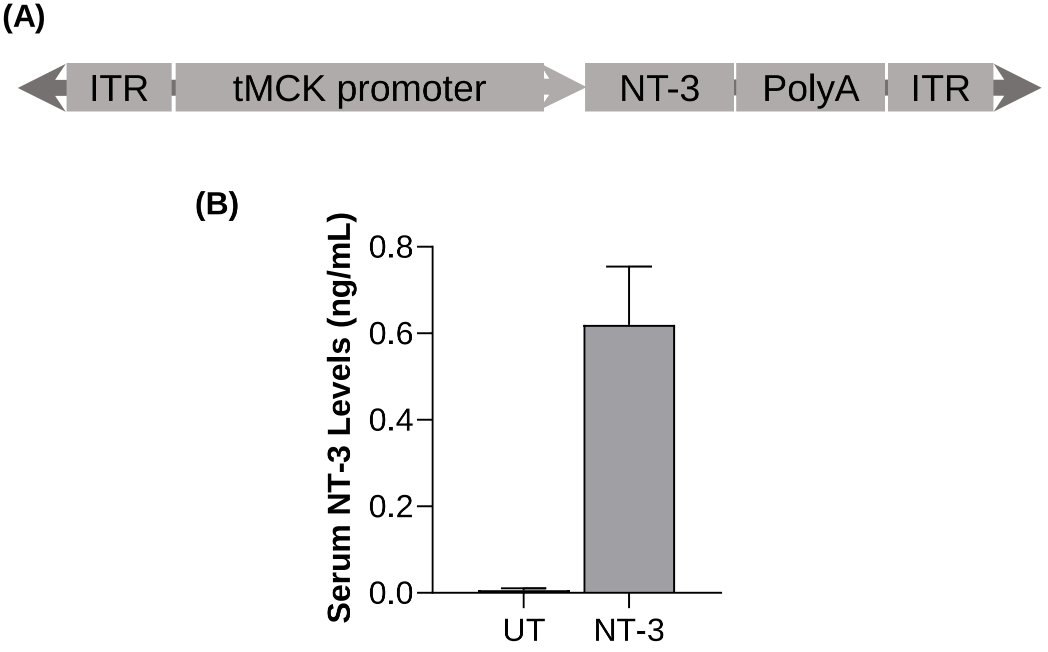

Figure S1. scAAV1.tMCK.NT-3 vector and serum NT-3 levels. *(A)* NT-3 expression is driven by a tMCK in the scAAV vector backbone. The diagram shows the cassette composed of a tMCK enhancer/ promoter region (714 bp), the full-length NT-3 cDNA (774 bp) and the SV40 polyA tail (211 bp); Ref#15. *(B)* Serum samples were collected from treated (NT-3) and untreated (UT) mice, and NT-3 levels were measured by ELISA. Error bars are ± SEM; n=5 for treated cohort, n=4 for untreated cohort. NT-3 serum levels were below detection range for untreated mice.

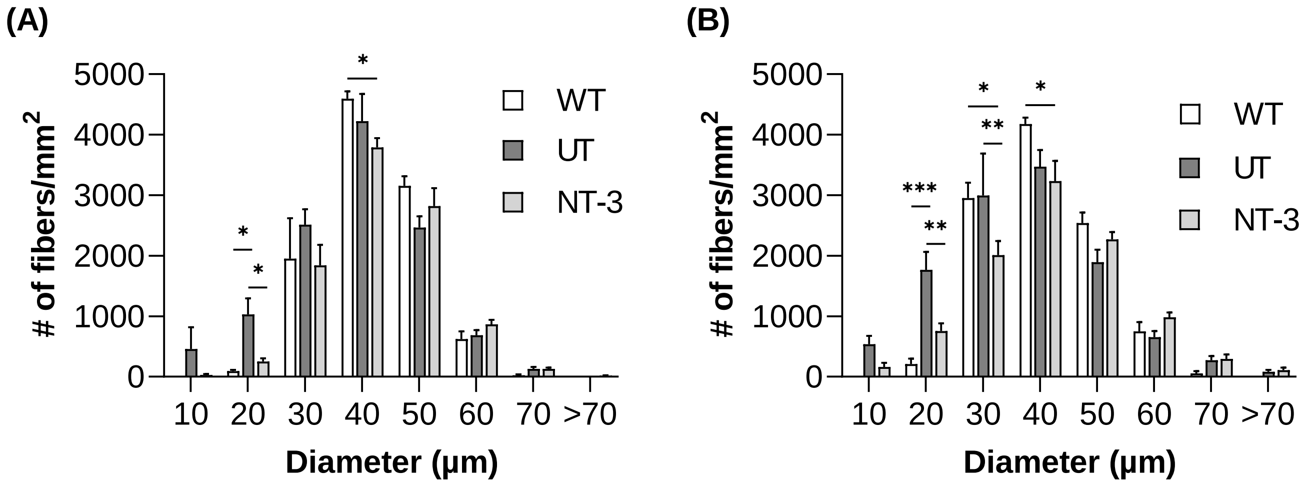

Figure S2. Fiber size distribution in treated and untreated SOD1KO mice and age-matched wild-type mice. *(A)* Tibialis anterior fiber size distribution (n=6, NT-3; n=6, UT; n=4, WT. *(B)* Gastrocnemius fiber size distribution (n=11, NT-3; n=7, UT; n=4, WT). Data are represented as mean ± SEM; *P<0.05, **P<0.01, ***P<0.001, two-way ANOVA, Tukey's multiple comparisons test.

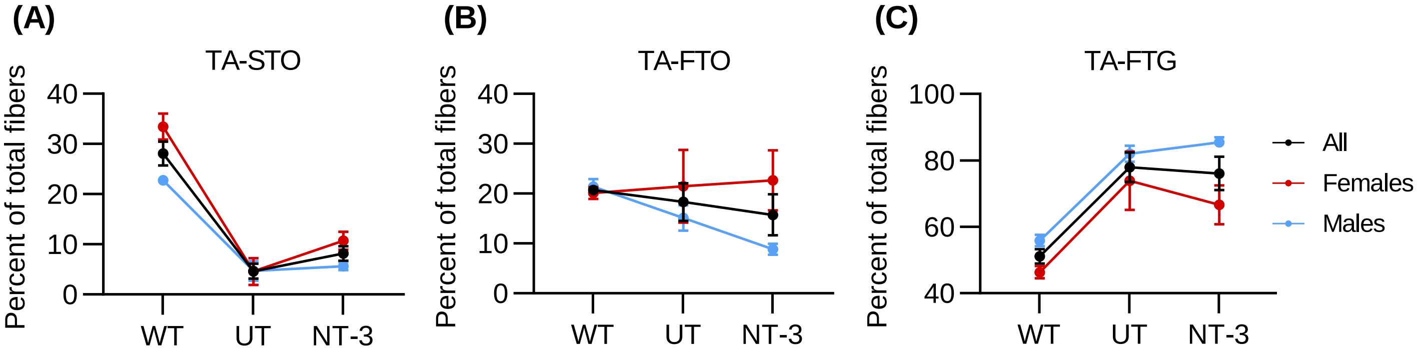

Figure S3. Graphs represent the changes in the percent distribution of *(A)* STO, *(B)* FTO and *(C)* FTG fiber types in the tibialis anterior muscle from UT and treated cohorts and age-matched WT mice, shown as sexes combined (black) and separated (blue for males, red for females). n=6, NT-3; n=6, UT; n=8, WT; even sex-distribution for all cohorts. Data are represented as mean ± SEM.

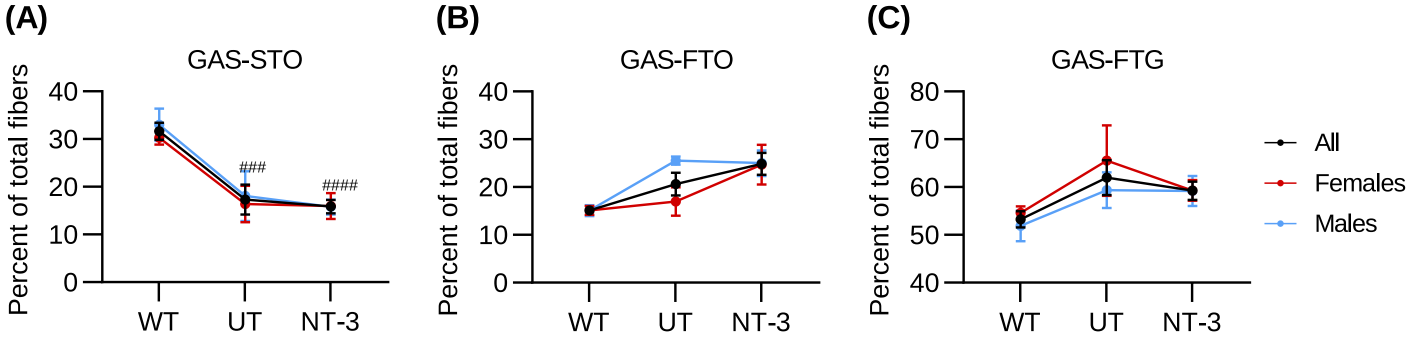

Figure S4. Graphs represent the changes in the percent distribution of *(A)* STO, *(B)* FTO and *(C)* FTG fiber types in the gastrocnemius muscle from UT and treated cohorts and age-matched WT mice, shown as sexes combined (black) and separated (blue for males, red for females). n=11, NT-3; n=7, UT; n=8, WT; even sex-distribution for all cohorts. Data are represented as mean ± SEM.

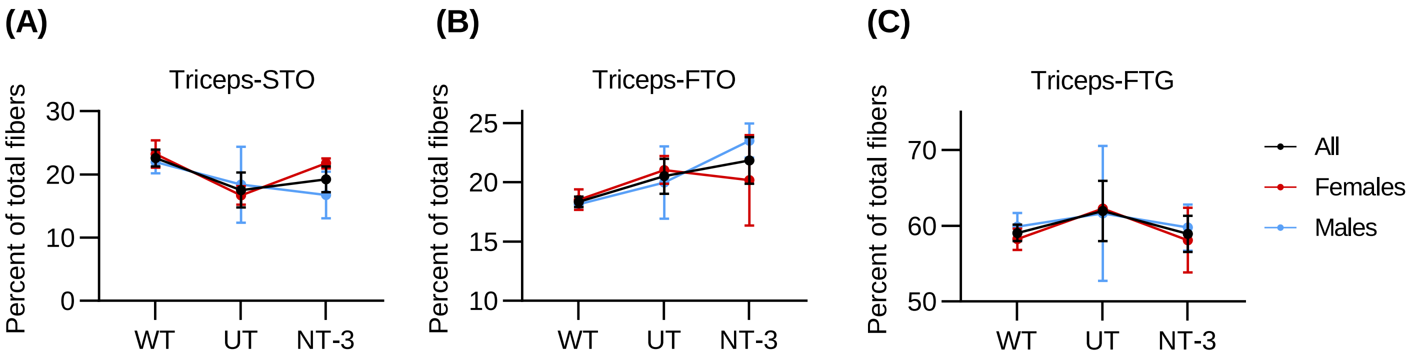

Figure S5. Graphs represent the changes in the percent distribution of *(A)* STO, *(B)* FTO and *(C)* FTG fiber types in the triceps muscle from UT and treated cohorts and age-matched WT mice, shown as sexes combined (black) and separated (blue for males, red for females). n=6, NT-3; n=6, UT; n=8, WT; even sex-distribution for all cohorts. Data are represented as mean ± SEM.

Table S1 Fiber size analysis on tibialis anterior muscle of NT-3 treated and untreated SOD1KO mice.

		WT (n=8)		Untreated (n=6)		NT-3 Treated (n=6)	
		Number	Diameter (µm)	Number	Diameter (µm)	Number	Diameter (µm)
ALL	STO	91.6 ± 10.1	30.49 ± 1.0	16.3 ± 5.5	30.58 ± 1.1	24.2 ± 4.5	31.91 ± 1.4
	FTO	66.3 ± 3.5	36.33 ± 1.1	62.3 ± 12.3	33.46 ± 1.8	46.0 ± 11.4	34.47 ± 1.3
	FTG	162.5 ± 5.7	41.54 ± 2.0	268.0 ± 19.2	33.84 ± 1.3$^{\#\#\#\#}$	223.3 ± 15.9	38.69 ± 0.8*
	All Fibers	320.4 ± 13.6	37.23 ± 0.9	346.7 ± 14.5	33.74 ± 1.2	293.5 ± 9.1	37.75 ± 0.8*

		WT (n=4)		Untreated (n=3)		NT-3 Treated (n=3)	
		Number	Diameter (µm)	Number	Diameter (µm)	Number	Diameter (µm)
Females	STO	118.3 ± 7.0	27.98 ± 0.5	15.3 ± 8.9	32.65 ± 0.1	32.3 ± 5.4	32.56 ± 2.4
	FTO	71.5 ± 4.8	34.61 ± 1.7	72.3 ± 24.7	36.49 ± 1.2	67.0 ± 14.3	33.68 ± 2.2
	FTG	165.3 ± 10.1	40.89 ± 1.2	247.3 ± 28.3	35.05 ± 0.6$^{\#}$	204.0 ± 28.0	37.14 ± 0.4
	All Fibers	355.0 ± 11.1	35.26 ± 0.9	335.0 ± 3.1	35.18 ± 0.4	303.3 ± 16.2	36.18 ± 0.4

		WT (n=4)		Untreated (n=3)		NT-3 Treated (n=3)	
		Number	Diameter (µm)	Number	Diameter (µm)	Number	Diameter (µm)
Males	STO	65.0 ± 1.8	33.00 ± 0.5	17.3 ± 8.4	28.52 ± 1.5	16.0 ± 2.1	31.26 ± 2.0
	FTO	61.0 ± 3.6	38.04 ± 0.9	52.3 ± 6.7	30.43 ± 2.2$^{\#\#}$	25.0 ± 2.6	35.26 ± 1.6
	FTG	159.8 ± 5.1	42.19 ± 0.5	288.7 ± 24.7	32.62 ± 2.6$^{\#\#\#}$	242.7 ± 10.2	40.23 ± 0.7**
	All Fibers	285.8 ± 3.8	39.20 ± 0.4	351.3 ± 23.6	32.30 ± 2.3$^{\#}$	283.7 ± 7.2	39.32 ± 0.6*

Data represented as mean ± SEM. *p<0.05, **p<0.01, (compared to UT), $^{\#}$p<0.05, $^{\#\#}$p<0.01, $^{\#\#\#}$p<0.001, $^{\#\#\#\#}$p<0.0001 (compared to WT), Two-way ANOVA for size analysis of fiber types, one-way ANOVA for total fiber size analysis, Tukey's multiple comparison test.

Table S2 Fiber size analysis on gastrocnemius muscle of NT-3 treated and untreated SOD1KO mice.

		WT (n=8)		Untreated (n=7)		NT-3 Treated (n=11)	
		Number	Diameter (µm)	Number	Diameter (µm)	Number	Diameter (µm)
ALL	STO	101.9 ± 6.8	29.96 ± 0.7	65.1 ± 18.0	30.07 ± 1.6	46.2 ± 6.0	31.86 ± 1.4
	FTO	48.5 ± 2.6	34.07 ± 0.7	71.4 ± 8.0	32.77 ± 2.0	73.0 ± 10.3	36.04 ± 1.1
	FTG	170.5 ± 6.3	41.19 ± 1.0	214.9 ± 14.1	32.53 ± 1.2####	176.6 ± 17.3	39.23 ± 1.9***
	All Fibers	320.9 ± 9.7	36.51 ± 0.8	351.4 ± 28.3	32.34 ± 1.2#	295.8 ± 20.3	37.18 ± 1.0*

		WT (n=4)		Untreated (n=4)		NT-3 Treated (n=5)	
		Number	Diameter (µm)	Number	Diameter (µm)	Number	Diameter (µm)
Females	STO	103.3 ± 5.2	28.69 ± 0.5	81.3 ± 26.0	27.41 ± 1.0	51.4 ± 9.3	30.27 ± 0.9
	FTO	53.7 ± 4.0	32.67 ± 0.3	64.0 ± 11.8	31.10 ± 2.6	79.2 ± 14.2	35.04 ± 0.4
	FTG	185.8 ± 4.0	39.17 ± 0.5	231.3 ± 16.1	31.81 ± 1.9###	190.6 ± 9.5	37.56 ± 0.8**
	All Fibers	340.8 ± 8.9	34.97 ± 0.5	376.5 ± 43.3	31.07 ± 2.0	321.2 ± 9.1	35.76 ± 0.6*

		WT (n=4)		Untreated (n=3)		NT-3 Treated (n=6)	
		Number	Diameter (µm)	Number	Diameter (µm)	Number	Diameter (µm)
Males	STO	100.5 ± 12.4	31.24 ± 0.9	43.7 ± 13.5	33.63 ± 1.2	41.8 ± 2.3	33.19 ± 1.5
	FTO	43.0 ± 3.0	36.32 ± 1.0	81.3 ± 4.3	34.99 ± 2.6	67.8 ± 8.2	36.88 ± 1.4
	FTG	155.3 ± 5.1	43.20 ± 1.3	193.0 ± 16.1	33.50 ± 0.8##	165.0 ± 21.8	40.63 ± 2.5*
	All Fibers	301.0 ± 9.9	38.04 ± 0.9	318.0 ± 8.5	34.04 ± 0.6	274.7 ± 23.9	38.35 ± 1.8

Data represented as mean ± SEM. *p<0.05, **p<0.01, ***p<0.001, (compared to UT), #p<0.05, ##p<0.01, ###p<0.001, ####p<0.0001 (compared to WT), Two-way ANOVA for size analysis of fiber types, one-way ANOVA for total fiber size analysis, Tukey’s multiple comparison test.

Table S3 Fiber size analysis on triceps muscle of NT-3 treated and untreated SOD1KO mice.

		WT (n=8)		Untreated (n=6)		NT-3 Treated (n=6)	
		Number	Diameter (µm)	Number	Diameter (µm)	Number	Diameter (µm)
ALL	STO	72.4 ± 5.2	26.22 ± 0.7	48.8 ± 6.0	25.84 ± 0.9	59.5 ± 9.2	26.14 ± 1.5
	FTO	58.8 ± 3.1	33.32 ± 0.7	58.3 ± 4.0	32.30 ± 1.0	67.3 ± 9.5	33.74 ± 2.0
	FTG	188.9 ± 8.8	43.50 ± 1.1	179.8 ± 18.0	44.31 ± 1.6	181.8 ± 17.4	43.15 ± 2.5
	All Fibers	320.0 ± 14.5	37.70 ± 0.8	287.0 ± 16.3	38.48 ± 0.9	308.7 ± 28.5	37.77 ± 2.1

		WT (n=4)		Untreated (n=3)		NT-3 Treated (n=3)	
		Number	Diameter (µm)	Number	Diameter (µm)	Number	Diameter (µm)
Females	STO	81.8 ± 7.0	25.88 ± 1.1	52.7 ± 5.6	24.08 ± 0.5	74.3 ± 8.6	25.12 ± 1.4
	FTO	65.3 ± 4.0	35.35 ± 0.5	66.0 ± 2.5	31.54 ± 0.2	70.0 ± 18.0	32.68 ± 1.6
	FTG	204.8 ± 9.5	41.22 ± 1.0	195.7 ± 3.3	41.95 ± 1.0	195.7 ± 9.6	40.68 ± 1.1
	All Fibers	351.8 ± 15.0	35.96 ± 1.0	314.3 ± 6.7	36.79 ± 0.7	340.0 ± 27.2	35.71 ± 1.6

		WT (n=4)		Untreated (n=3)		NT-3 Treated (n=3)	
		Number	Diameter (µm)	Number	Diameter (µm)	Number	Diameter (µm)
Males	STO	63.0 ± 4.0	26.57 ± 0.8	45.0 ± 11.6	27.60 ± 0.8	44.7 ± 11.3	27.17 ± 2.9
	FTO	52.3 ± 1.5	34.29 ± 1.1	50.7 ± 3.8	33.07 ± 2.2	64.7 ± 11.0	34.81 ± 4.0
	FTG	173.0 ± 9.6	45.77 ± 1.2	164.0 ± 37.0	46.66 ± 2.6	168.0 ± 35.1	45.62 ± 4.9
	All Fibers	288.3 ± 10.4	39.45 ± 0.6	259.7 ± 23.0	40.17 ± 0.7	277.3 ± 48.3	39.83 ± 3.9

Data represented as mean ± SEM. Two-way ANOVA for size analysis of fiber types, one-way ANOVA for total fiber size analysis, Tukey’s multiple comparison test.

Table S4 Average G ratios of the tibial and sciatic nerves from SOD1KO mice.

Nerve	Cohorts	n	Number of measurements	Mean ± SEM
TIBIAL	Untreated	4	1982	0.721 ± 0.002
	NT-3 Treated	4	1839	0.646 ± 0.002
	WT	4	1658	0.665 ± 0.002
SCIATIC	Untreated	6	3377	0.676 ± 0.001
	NT-3 Treated	6	2834	0.634 ± 0.001
